# Microwave Accelerated Aza-Claisen Rearrangement

**DOI:** 10.3390/molecules131102837

**Published:** 2008-11-14

**Authors:** Eva Gajdošíková, Miroslava Martinková, Jozef Gonda, Patrik Čonka

**Affiliations:** Institute of Chemical Sciences, Department of Organic Chemistry, P. J. Šafárik University, Moyzesova 11, SK-040 01 Košice, Slovak Republic; E-mails: eva.gajdosikova@gmail.com (E. G.), miroslava.martinkova@upjs.sk (M. M.), patrik.conka@gmail.com (P. C.)

**Keywords:** Overman rearrangement, Imidate, Microwave irradiation

## Abstract

A study of microwave-induced and standard thermal Overman rearrangement of selected allylic trichloroacetimidates **1a**-**1f**, **6-8** to the corresponding acetamides **2a**-**2f**, **9- 11** is reported. The microwave-assisted rearrangement of trifluoroacetimidate **13** is also described. Using this methodology, an efficient access to versatile allylic trihaloacetamides building synthons was established.

## Introduction

The [3,3]-sigmatropic rearrangement of allylic trihaloacetimidates into allylic trihaloacetamides is a useful methodology for the synthesis of nitrogen containing compounds such as amino acids [1,2,3], modified nucleosides [4,5] or other complex biologically interesting products [6,7,8,9]. This transformation is very often involved as the key step in the synthetic approaches and can be accomplished either at elevated temperatures or catalyzed by metal salts such as Hg(OCOCF_3_)_2_ [10,11], PdCl_2_ complexes [12,13] and new Pt(II), Pt(IV), Au(I) and Au(III) catalysts [14] under very mild reaction conditions.

A significant acceleration of aza-Claisen rearrangements was observed using microwave irradiation [15]. This fact eliminated problems with previously required high temperatures and extended reaction times, and also reduced decomposition of the starting materials and products. 

## Results and Discussion

In this **communicatio**n, we wish to report on microwave-assisted thermal Overman rearrangement of some selected allylic trihaloacetimidates **1a-f**, **6-8, 13** that are derived either from simple allylic alcohols, amino acids or the modified sugars, respectively, and thus illustrate the potential of microwave irradiation to accelerate this reaction. 

**Scheme 1:**
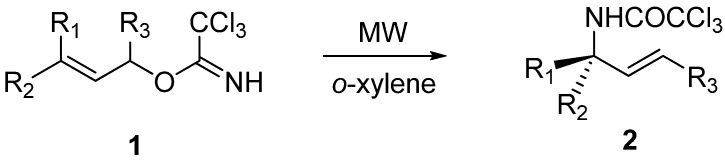
Microwave accelerated Overman rearrangement of simple aliphatic imidates.

**Table 1:** Microwave accelerated Overman rearrangement of simple aliphatic imidates.

Entry	R	Conditions	Time	Yield^a^ (%) 2a-f
1	R^1^= H, R^2^= H, R^3^= H **(a)**	Δ, 140 ^o^C, *o*-xylene [10,15]	12 h	50
		MW, 140 ^o^C, *o*-xylene, K_2_CO_3_	10 h	65
		MW, 210 ^o^C, *o*-xylene, K_2_CO_3_^b^	5 min	70
2	R^1^= H, R^2^= H, R^3^= Me **(b)**	Δ, 110^o^C, toluene [18]	2 h	53
		Δ, 140 ^o^C, *o*-xylene, K_2_CO_3_	2 h	60
		MW, 140 ^o^C, *o*-xylene, K_2_CO_3_ [16]	15 min	93
		MW, 180 ^o^C, *o*-xylene, K_2_CO_3_^b^ [16]	8 min	89
3	R^1^= H, R^2^= H, R^3^= Bu **(c)**	Δ, 140 ^o^C, *o*-xylene [10]	2.5 h	74
		Δ, 140 ^o^C, *o*-xylene, K_2_CO_3_	2.5 h	75
		MW, 140 ^o^C, *o*-xylene, K_2_CO_3_ [16]	5 min	97
		MW, 180 ^o^C, *o*-xylene, K_2_CO_3_^b^ [16]	1 min	94
4	R^1^= H, R^2^= H, R^3^= Pent **(d)**	Δ, 140 ^o^C, *o*-xylene, K_2_CO_3_	1.5 h	97
		MW, 140 ^o^C, *o*-xylene, K_2_CO_3_	5 min	92
5	R^1^= Me, R^2^= Me, R^3^= H **(e)**	Δ, 140 ^o^C, *o*-xylene [11]	3.5 h	48
		Δ, 140 ^o^C, *o*-xylene, K_2_CO_3_	3.5 h	80
		MW, 140 ^o^C, *o*-xylene, K_2_CO_3_ [16]	20 min	85
		MW, 180 ^o^C, *o*-xylene, K_2_CO_3_^b^ [16]	14 min	84
6	R^1^= Me, R^2^= H, R^3^= Me **(f)**	Δ, 140 ^o^C, *o*-xylene, K_2_CO_3_	45 min	70
		MW, 140 ^o^C, *o*-xylene, K_2_CO_3_	45 min	75

^a^Isolated yield. ^b^MW experiments were performed in the presence of a heating bar, Weflon, Milestone.

Thermally driven [3,3]-sigmatropic rearrangements (Scheme 1) were carried out according to the procedure described by Overman [10]. In the microwave-assisted thermal aza-Claisen rearrangement, the imidate was dissolved in *o*-xylene, powdered anhydrous K_2_CO_3_ [17] (2 mg/mL) was added, and the solution was heated under sealed vessel conditions. The scope of this method was investigated and all synthesized imidates (only imidates **1e** and **1f** were not characterized and used immediately to avoid problems connected with their instability) in Table 1 were converted to the corresponding trichloroacetamides **2a**–**2f** in considerably shorter reaction times, compared to the conventional thermal rearrangement. We have observed that the use of microwave irradiation lead to a substantial reduction of the reaction times (from hours to minutes, Table 1, Entry 1-5). On the other hand, the conversion of **1f** to compound **2f** was achieved at the same reaction time in the both cases (the microwave-assisted and standard thermal conditions, Table 1, Entry 6). 

**Scheme 2:**
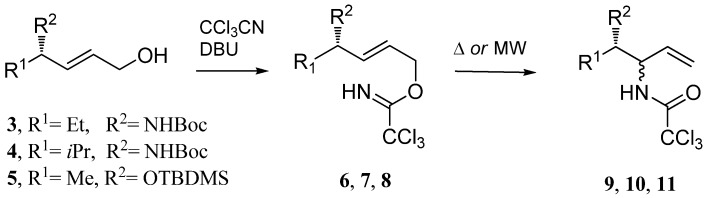
Microwave accelerated Overman rearrangement of the chiral imidates.

**Table 2:** Microwave accelerated Overman rearrangement of the chiral imidates **6**, **7**, **8** and **13**.

Entry	Starting material	Conditions	Time	Yield^a^ (%)
1	**6**	Δ, 140 ^o^C, *o*-xylene, *de*=10% [12]	24 h	75
		MW, 140 ^o^C, *o*-xylene, K_2_CO_3_ *de*=14% [16]	2 h	80
		MW, 200 ^o^C, *o*-xylene, K_2_CO_3_ *de*=12% [16]	5 min	80
2	**7**	Δ, 140 ^o^C, *o*-xylene, *de*=10% [12]	24 h	69
		MW, 140 ^o^C, *o*-xylene, K_2_CO_3_ *de*=12% [16]	2 h	71
		MW, 200 ^o^C, *o*-xylene, K_2_CO_3_ *de*=13% [16]	5 min	68
3	**8**	Δ, 140 ^o^C, *o*-xylene, *de*=2%	42 h	80
		MW, 160 ^o^C, *o*-xylene, K_2_CO_3_ *de*=2%	1 h	86
4	**13**	Δ, 180 °C, *o*-xylene, K_2_CO_3_, *de* ≈ 20%	12 h	31
		MW, 180 °C, *o*-xylene, K_2_CO_3_, *de* ≈ 19%	30 min	68
		MW, 180 °C, *o*-xylene, *de* ≈ 18%	1 h	15

^a^Isolated yields.

In earlier studies was found that Pd(II)-catalyzed Overman rearrangement of trichloroacetimidates **6**, **7** derived from primary allylic alcohols with an adjacent centre of chirality proceeded with an excellent diastereoselectivity (*de* ≥ 98%) [12]. In the next phase of our work we decided to study whether the described microwave-assisted Overman rearrangement could lead to a certain degree of diastereoselection. The conversion of known allylic alcohols [12,14] into trichloroacetimidates **6-8** was achieved using trichloroacetonitrile and DBU as a base in dichloromethane (Scheme 2). The results of the thermal and microwave induced Overman rearrangements of imidates **6-8** are summarized in Table 2. We have found that microwave irradiation of **6- 8** led to the rearranged products **9**, **10 [12]** and **11** [14] (as the mixtures of diastereoisomers) with substantial shortening of the reaction times (from 24 h to 5 min) with good yields (Table 2), however, it has shown that in these cases microwave-induced rearrangement had practically no influence on the diastereoselectivity of aza-Claisen rearrangement (Table 2). 

**Scheme 3:**
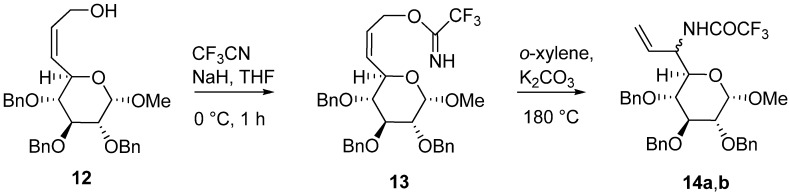
Microwave accelerated Overman rearrangement of the sugar trifluoroacetimidate **13**.

Finally, we have investigated Overman rearrangement of the sugar allylic trifluoroacetimidate **13** under microwave irradiation. Trifluoroacetimidate **13** was prepared from the corresponding allylic alcohol **12** derived from d-glucose [19] by reaction with CF_3_CN in THF (Scheme 3). Rearrangement of **13** afforded trifluoroacetamide **14** as the mixture of diastereoizomers (*de*=19%) (Scheme 3). In order to determine the best reaction conditions, a series of the thermally and microwave accelerated rearrangements of imidate **13** was performed. Studies showed that microwave irradiation accelerated of the rearrangement **13**→**14a,b** (24 times) in comparison with conventional thermal conditions (Table 2, entry 4) without any improvement in the stereoselectivity. Extension of the reaction time led to the decomposition of product **14**.

## Conclusions

In summary, a remarkable acceleration of the Overman rearrangemet of allylic trihaloimidates to the corresponding allylic trihaloamides was observed using microwave irradiation conditions. The [3,3]-sigmatropic rearrangement carried out under conventional conditions (reflux temperature of the solvent) required long reaction times and produced moderate yields, usually a result of connected with the decomposition of starting materials. This paper demonstrates the practical usability of microwave acceterated thermal Overman rearrangement for the synthesis of various amides.

## Experimental

All commercially available reagents were used without further purification and solvents were dried according to standard procedures. Column chromatography was carried out on Silica Gel 60 (Merck, 0.040-0.063 mm, 230-400 mesh). Analytical thin-layer chromatography (TLC) was performed on Merck silica gel 60 F_254_ analytical plates; detection was carried out with either UV (254 nm), or spraying with a solution of phosphomolybdic acid, and with a basic solution of KMnO_4_, with subsequent heating. NMR spectra were recorded at room temperature on a Varian Mercury Plus 400 FT NMR spectrometer (^1^H at 400.13 MHz and ^13^C at 100.6 MHz), in CDCl_3_ as the solvent (unless otherwise noted) with tetramethylsilane as internal reference. For those fully assigned ^1^H- and ^13^C-NMR spectra standard NMR (COSY, DEPT, HSQC) experiments were conducted. Optical rotations were measured with a P3002 Krüss polarimeter in chloroform at 25 ^o^C. All moisture-sensitive reactions were performed under a nitrogen atmosphere. Microwave experiments were conducted using a focused microwave system (CEM Discover). Reactions were performed in a glass vessel (10 mL) sealed with a septum. At the end of the reaction the vessels together with their contents were cooled rapidly using a stream of compressed air. The melting points were determined on the Kofler block and are uncorrected.

### General procedure for preparation of trichloroacetimidates

To a solution of allyl alcohol in dry dichloromethane were added 1,8-diazabicyclo[5,4,0]undec-7-ene (DBU, 2 eq) and trichloroacetonitrile (2 eq) at 0 ^o^C. The reaction mixture was stirred at 0 ^o^C for 1 h. The insoluble material was removed by filtration and the filtrate was concentrated under reduced pressure to give a residue, which was purified by chromatography on silica gel (cyclohexane-ethyl acetate) to afford corresponding imidates **1a**-**1d**, **6**, **7**, **8**. 

*O-Allyl-2,2,2-trichloroacetimidate*: Allyl alcohol (0.50 g, 8.61 mmol), DBU (2.57 mL, 17.22 mmol), trichloroacetonitrile (1.73 mL, 17.22 mmol) in CH_2_Cl_2_ (20 mL) afforded after flash chromatography (cyclohexane-ethyl acetate, 10:1) compound **1a** (1.56 g, 89.5%) as a colorless oil; ^1^H-NMR: δ 4.81 (2H, m, CH_2_), 5.31 (1H, dd, *J*=10.5 Hz, *J*=1.3 Hz, CH_2_=), 5.44 (1H, ddd, *J*=17.2 Hz, *J*=3.1 Hz, *J*=1.5 Hz, CH_2_=), 6.03 (1H, ddd, *J*=17.2 Hz, *J*=10.5 Hz, *J*=5.4 Hz, CH=), 8.32 (1H, bs, NH); ^13^C-NMR: δ 69.6, 109.7, 118.5, 131.4, 162.5; Anal. Calcd. for C_5_H_6_Cl_3_NO (202.47): C 29.66, H 2.99, N 6.91; found C 29.53, H 2.87, N 6.74. The procedure and ^1^H-NMR spectroscopic data were previously reported [10]. ^13^C-NMR data have not been reported before [10].

*O-(But-3-en-2-yl)-2,2,2-trichloroacetimidate* (**1b**): But-3-en-2-ol (0.50 g, 6.93 mmol), DBU (1.45 mL, 9.70 mmol, 1.4 eq), trichloroacetonitrile (1.04 mL, 10.4 mmol, 1.5 eq) in CH_2_Cl_2_ (25 mL) afforded (1.30 g, 87%) of compound **1b** after flash chromatography (cyclohexane-ethyl acetate, 5:1) as a pale yellow oil; ^1^H-NMR: δ 1.44 (3H, d, *J*=6.5 Hz, CH_3_), 5.20 (1H, d, *J*=10.6 Hz, H_4_), 5.36 (1H, d, *J*=17.3 Hz, H_4_), 5.49 (1H, m, H_3_), 5.94 (1H, m, H_2_), 8.29 (1H, bs, NH); ^13^C-NMR: δ 19.4, 75.7, 91.8, 115.9, 136.8, 161.8.; Anal. Calcd. for C_6_H_8_Cl_3_NO (216.49): C 33.29, H 3.72, N 6.47; found C 33.10, H 3.45, N 6.28. The procedure and ^1^H-NMR spectroscopic data have been reported [18]. ^13^C-NMR data have not previously been reported [18].

*2,2,2-Trichloro-O-(hept-1-en-3-yl)acetimidate* (**1c**): Hept-1-en-3-ol (0.50 g, 4.38 mmol), DBU (1.31 mL, 8.76 mmol), trichloroacetonitrile (0.88 mL, 8.76 mmol) in CH_2_Cl_2_ (20 mL) afforded after flash chromatography (cyclohexane-ethyl acetate, 10:1) compound **1c** (1.11 g, 98%) as a pale yellow oil; ^1^H-NMR: δ 0.90 (3H, t, *J*=6.9 Hz, CH_3_), 1.38 (4H, m, 2 x CH_2_), 1.74 (2H, m, CH_2_), 5.21 (1H, dd, *J*_2,1_=10.6 Hz, *J*_1,1_=0.7 Hz, H_1_), 5.36 (2H, m, H_1_, H_3_), 5.80 (1H, m, H_2_), 8.27 (1H, s, NH); ^13^C-NMR: δ 14.2, 22.6, 27.3, 34.0, 79.7 92.1, 116.8, 135.8, 162.2; Anal. Calcd for C_9_H_14_Cl_3_NO_2_ (258.57): C 41.76, H 5.41, N 5.41; found C 41.65, H 5.21, N 5.33. The procedure and ^1^H-NMR spectroscopic data were reported [10]. ^13^C-NMR data have not previously been reported [10].

*2,2,2-Trichloro-O-(oct-1-en-3-yl)acetimidate* (**1d**): Oct-1-en-3-ol (0.50 g, 3.90 mmol), DBU (1.17 mL, 7.8 mmol), trichloroacetonitrile (0.78 mL, 7.80 mmol) in CH_2_Cl_2_ (20 mL) afforded after flash chromatography (cyclohexane-ethyl acetate, 10:1) compound **1d** (0.90 g, 85%) as a pale yellow oil; ^1^H-NMR: δ 0.88 (3H, t, *J*=7.1 Hz, CH_3_), 1.29-1.47 (6H, m, 3 x CH_2_), 1.65-1.82 (2H, m, CH_2_), 5.21 (1H, m, H_1_), 5.30 (2H, m, H_1_, H_3_), 5.86 (1H, m, H_2_), 8.26 (1H, bs, NH); ^13^C-NMR: δ 14.2, 79.7, 22.7, 24.8, 31.7, 34.3, 92.1, 116.7, 136.0, 162.2; Anal. Calcd. for C_10_H_16_Cl_3_NO (272.60): C 44.06, H 5.91, N 5.14; found C 43.95, H 5.77, N 5.01.

*tert-Butyl N-[(3S,4E)-6-(trichloroacetimidyloxy)hex-4-en-3-yl]carbamate* (**6**): Compound **3** (0.30 g, 1.393 mmol), DBU (0.42 mL, 2.79 mmol), trichloroacetonitrile (0.28 mL, 2.79 mmol) in CH_2_Cl_2_ (15 mL) afforded after flash chromatography (cyclohexane-ethyl acetate, 3:1) compound **6** (0.40 g, 80%) as a colorless oil; ^1^H-NMR: δ 0.92 (3H, t, *J*=7.4 Hz, CH_3_), 1.44 (9H, s, 3 x CH_3_), 1.53 (2H, m, CH_2_), 4.08 (1H, m, H_3_), 4.45 (1H, bs, NH), 4.77 (2H, m, H_6_), 5.79 (2H, m, H_4_, H_5_), 8.29 (1H, bs, NH); ^13^C-NMR: δ 10.1, 28.2, 28.4 (3x), 53.1, 68.9, 79.4, 123.4, 135.8, 155.4, 162.5; Anal. Calcd. for C_13_H_21_Cl_3_N_2_O_3_ (359.68): C 43.37, H 5.83, N 7.78; found C 43.01, H 5.64, N 7.64. ^1^H and ^13^C-NMR spectroscopic data have not previously been reported [12].

*tert-Butyl N-[(3S,4E)-6-(trichloroacetimidyloxy)-2-methylhex-4-en-3-yl]carbamate* (**7**): Compound **4** (0.10 g, 0.436 mmol), DBU (0.13 mL, 0.87 mmol), trichloroacetonitrile (0.087 mL, 0.87 mmol) in CH_2_Cl_2_ (10 mL) afforded after flash chromatography (cyclohexane-ethyl acetate, 3:1) compound **7** (0.12 g, 74%) as white crystals; m.p. 42 – 43 ^o^C; ^1^H-NMR: δ 0.89 (6H, m, 2 x CH_3_), 1.44 (9H, s, 3 x CH_3_), 1.78 (1H, m, CH), 4.04 (1H, m, H_3_), 4.52 (1H, m, NH), 4.80 (2H, m, H_6_), 5.79 (2H, m, H_4_, H_5_), 8.30 (1H, bs, NH); ^13^C-NMR: δ 18.1, 18.7, 28.4, (3 x C), 32.4, 56.9, 68.9, 79.4, 91.4, 123.9, 134.5, 155.5, 162.4; Anal. Calcd. for C_14_H_23_Cl_3_N_2_O_3_ (373.71): C 44.99, H 6.20, N 7.49; found C 44.78, H 6.05, N 7.21. ^1^H- and ^13^C-NMR spectroscopic data have not previously been reported [12].

*O-[(4S,2E)-4-(tert-Butyldimethylsilyoxy)pent-2-enyl]-2,2,2-trichloroacetimidate* (**8**): Compound **5** (0.35 g, 1.62 mmol), DBU (0.48 mL, 3.24 mmol), trichloroacetonitrile (0.325 mL, 3.24 mmol) in CH_2_Cl_2_ (18 mL) afforded compound **8** (0.50 g, 85.5%) as a colorless oil; ^1^H-NMR: δ 0.05 (3H, s, CH_3_), 0.06 (3H, s, CH_3_), 0.89 (9H, s, 3 x CH_3_), 1.23 (3H, d, *J*=6.7 Hz, CH_3_), 4.35 (1H, m, H_4_), 4.77 (2H, m, H_1_), 5.86 (2H, m, H_2_, H_3_), 8.28 (1H, bs, NH); ^13^C-NMR: δ -4.8, -4.7, 18.3, 24.1, 25.9 (3 x C), 68.4, 69.1, 121.4, 139.8, 162.5; Anal. Calcd. for C_13_H_24_Cl_3_NO_2_Si (360.78): C 43.28, H 6.71, N 3.88; found C 43.17, H 6.56, N 3.59. The procedure and ^1^H-NMR spectroscopic data were reported before [14]. ^13^C-NMR data have not previously been reported [14].

### General procedure for Overman rearrangement

*Conventional method (Procedure A):* To a solution of imidates in dry solvent (see Table 1 and Table 2) was added anhydrous K_2_CO_3_ (1.1 eq). The reaction mixture was heated (for temperatures see Table 1 and Table 2). The solvent was evaporated under reduced pressure and chromatography of the residue on the silica gel (cyclohexane-ethyl acetate) afforded corresponding amides **2a**-**2f**, **9-11, 14** (Table 1 and Table 2). (*B1*)

*Microwave-assisted synthesis (Procedure B)*: To a solution of the corresponding imidate in *o*-xylene in a 10 mL glass pressure microwave tube equipped with a magnetic stirrer bar was added anhydrous K_2_CO_3_ (1.1 eq) and the tube was closed with a silicon septum. The reaction mixture was subjected to microwave irradiation (power: 300W; for temperatures, reaction times and yields see Table 1 and Table 2). The solvent was removed under reduced pressure and the residue was purified by flash chromatography on silica gel (cyclohexane-ethyl acetate) to give amides **2a**-**2f**, **9-11, 14** (Table 1 and Table 2). (*B2*)

*N-Allyl-2,2,2-trichloroacetamide* (**2a**): Following general procedure A, **1a** (0.30 g, 1.48 mmol), K_2_CO_3_ (0.23 g, 1.63 mmol) in *o*-xylene (3 mL) afforded after flash chromatography (cyclohexane-ethyl acetate, 10:1) compound **2a** (0.195 g, 65%). **2a**: white crystals; m.p. 28 - 32 ^o^C (Ref. [10] m.p. 28–31 ^o^C); ^1^H-NMR: δ 3.99-4.02 (2H, m, CH_2_), 5.24-5.32 (2H, m, CH_2_=), 5.84-5.93 (1H, m, CH=), 6.78 (1H, bs, NH); ^13^C-NMR: δ 43.6, 92.5, 117.8, 132.2, 161.8; Anal. Calcd. for C_5_H_6_Cl_3_NO (202.46): C 29.66, H 2.98, N 7.90; found C 29.59, H 2.83, N 7.75. The procedure and ^1^H-NMR spectroscopic data were reported [10]. ^13^C-NMR data have not previously been reported [10].

*N-[(E)-But-2-enyl]-2,2,2-trichloroacetamide* (**2b**): Following general procedure A, **1b** (0.10 g, 0.462 mmol), K_2_CO_3_ (70.2 mg, 0.508 mmol) in *o*-xylene (2 mL) afforded after flash chromatography (cyclohexane-ethyl acetate, 10:1) compound **2b** (0.093 g, 93%). Following general procedure B, **1b** (0.30 g, 1.386 mmol), K_2_CO_3_ (0.21 g, 1.525 mmol) in *o*-xylene (5 mL) afforded after flash chromatography (cyclohexane-ethyl acetate, 10:1) compound **2b** (0.18 g, 60%). **2b**: white crystals; m.p. 27 - 29 ^o^C (Ref. [10] m.p. 28 – 29 ^o^C); ^1^H-NMR: δ 1.72 (3H, d, *J*=6.5 Hz, CH_3_), 3.91 (2H, m, CH_2_), 5.50 (1H, m, CH=), 5.74 (1H, m, CH=), 6.68 (1H, bs, NH); ^13^C-NMR: δ 17.7, 43.3, 109.7, 124.8, 130.3, 161.6; Anal. Calcd. for C_6_H_8_Cl_3_NO (216.49): C 33.26, H 3.72, N 6.47; found C 33.14, H 3.55, N 6.38. The procedure, ^1^H-NMR and ^13^C-NMR data spectroscopic data have been reported [18]. 

*2,2,2-Trichloro-N-[(E)-hept-2-enyl]acetamide* (**2c**): Following general procedure A, **1c** (0.20 g, 0.773 mmol), K_2_CO_3_ (0.117 g, 0.85 mmol) in *o*-xylene (2 mL) afforded after flash chromatography (cyclohexane-ethyl acetate, 10:1) compound **2c** (0.18 g, 90%). Following general procedure B, **1c** (0.40 g, 1.55 mmol), K_2_CO_3_ (0.236 g, 1.71 mmol) in *o*-xylene (4 mL) afforded after flash chromatography (cyclohexane-ethyl acetate, 10:1) compound **2c** (0.30 g, 75%). **2c**: a colorless oil; ^1^H-NMR: δ 0.90 (3H, t, *J*=7.1 Hz, CH_3_), 1.28-1.39 (4H, m, 2 x CH_2_), 2.02-2.07 (2H, m, CH_2_), 3.92 (2H, m, CH_2_); 5.44-5.51 (1H, m, CH=), 5.68-5.76 (1H, m, CH=), 6.67 (1H; bs, NH); ^13^C-NMR: δ 14.1, 22.4, 31.3, 32.1, 43.6, 92.8, 123.7, 135.9, 161.8; Anal. Calcd. for C_9_H_14_Cl_3_NO_2_ (258.57): C 41.77, H 5.45, N 5.41; found C 41.63, H 5.24, N 5.35. The procedure and ^1^H-NMR spectroscopic data were reported before [10]. ^13^C-NMR data have not previously been reported [10].

*(E)-2,2,2-Trichloro-N-(oct-2-enyl)acetamide* (**2d**): Following general procedure A, **1d** (0.40 g, 1.47 mmol), K_2_CO_3_ (0.224 g, 1.62 mmol) in *o*-xylene (4 mL) afforded after flash chromatography (cyclohexane-ethyl acetate, 10:1) compound **2d** (0.37 g, 92.5%). Following general procedure B, **1d** (0.40 g, 1.47 mmol), K_2_CO_3_ (0.224 g, 1.62 mmol) in *o*-xylene (4 mL) afforded after flash chromatography (cyclohexane-ethyl acetate, 10:1) compound **2d** (0.39 g, 97.5%). **2d**: a colorless oil; ^1^H-NMR: δ 0.87 (3H, t, *J*=7.0 Hz, CH_3_), 1.26-1.40 (6H, m, 3 x CH_2_), 2.04 (2H, q, *J*=7.0 Hz, CH_2_), 3.93 (2H, t, *J*=5.9 Hz, CH_2_), 5.46 (1H, m, CH=), 5.73 (1H, m, CH=), 6.69 (1H, bs, NH); ^13^C-NMR: δ 14.6, 22.7, 28.8, 31.6, 32.4, 43.5, 92.8, 123.6, 135.8, 161.7; Anal. Calcd. for C_10_H_16_Cl_3_NO (272.60): C 44.02, H 5.92, N 5.13; found C 43.90, H 5.84, N 5.04. 

*2,2,2-Trichloro-N-(2-methylbut-3-en-2-yl)acetamide* (**2e**): Following general procedure A, **1e** (0.3 g, 1.30 mmol), K_2_CO_3_ (0.198 g, 1.43 mmol) in *o*-xylene (3 mL) afforded after flash chromatography (cyclohexane-ethyl acetate, 10:1) compound **2e** (0.25 g, 83%). Following general procedure B, **1e** (0.5g, 2.17 mmol), K_2_CO_3_ (0.33 g, 2.39 mmol) in *o*-xylene (5 mL) afforded after flash chromatography (cyclohexane-ethyl acetate, 10:1) compound **2e** (0.40 g, 80%). **2e**: white crystals; m.p. 48 – 50 ^o^C (Ref. [11] m.p. 49 – 50 ^o^C); ^1^H-NMR: δ 1.54 (6H, s, 2 x CH_3_), 5.17 (2H, m, H_4_), 6.00 (1H, m, H_3_), 6.59 (1H, bs, NH); ^13^C-NMR: δ 26.5 (2 x C), 56.1, 93.4, 113.3, 142.1, 160.4; Anal. Calcd. for C_7_H_10_Cl_3_NO (230.52): C 36.43, H 4.32, N 6.07, found C 36.30, H 4.11, N 5.98. The ^1^H-NMR spectrum was previously reported [11]. 

*2,2,2-Trichloro-N-[(E)-pent-3-en-2-yl]acetamide* (**2f**): Following general procedure A, **1f** (0.10 g, 0.434 mmol), K_2_CO_3_ (66 mg, 0.48 mmol) in *o*-xylene (2 mL) afforded after flash chromatography (cyclohexane-ethyl acetate, 10:1) compound **2f** (75 mg, 75%). Following general procedure B, **1f** (66 mg, 0.26 mmol), K_2_CO_3_ (43.5 mg, 0.315 mmol) in *o*-xylene (1 mL) afforded after flash chromatography (cyclohexane-ethyl acetate, 10:1) compound **2f** (46 mg, 70%) **2f**: white crystals; m.p. 57 – 59 ^o^C (Ref. [20] m.p. 60 ^o^C); ^1^H-NMR (DMSO [20]): δ 1.31 (3H, d, *J* =6.8 Hz, CH_3_), 1.71 (3H, m, CH_3_), 4.43-4.50 (1H, m, H_2_), 5.43-5.49 (1H, m, CH=), 5.65-5.75 (1H, m, CH=), 6.52 (1H, bs, NH); ^13^C-NMR (DMSO [20]): δ 17.9, 20.4, 49.0, 93.0, 127.6, 130.76, 161.03; Anal. Calcd. for C_7_H_10_Cl_3_NO (230.52): C 36.44, H 4.37, N 6.07, found C 36.35, H 4.21, N 5.97.

*Methyl (Z)-2,3,4-tri-O-benzyl-6,7-dideoxy-8-(trifluoroacetimidyloxy)-α-d-gluco-oct-6-enpyranoside* (**13**): To a suspension of NaH (0.09 g, 2.244 mmol, 60% dispersion in mineral oil, freed of oil with anhydrous THF) in dry THF (3 mL) was added allylic alcohol **12** (1.0 g, 2.04 mmol) in dry THF (5 mL) at 0 °C. The reaction mixture was stirred at 0 °C for 10 min and then treated with gaseous trifluoroacetonitrile (15 g, 0.158 mol, prepared *in situ* by heating trifluoroacetamide (4.57 g, 0.040 mol) and P_2_O_5_ (11.43 g, 0.102 mol) for 2 h at 150 °C). The solid was removed by filtration and solvent evaporated under reduced pressure. The residue was purified by chromatography on silica gel (hexane-ethyl acetate, 3:1) to afford 0.95 g (79.5%) of compound **13** as a pale yellow oil; ^1^H-NMR: δ 3.27 (1H, dd, *J*_4,3_=9.6 Hz, *J*_5,4_=9.1 Hz, H_4_), 3.40 (3H, s, OCH_3_), 3.52 (1H, dd, *J*_3,2_=9.7 Hz, *J*_2,1_=3.6 Hz, H_2_), 3.99 (1H, dd, *J*_3,2_=9.7 Hz, *J*_4,3_=9.6 Hz, H_3_), 4.45 (1H, ddd, *J*_5,4_=9.1 Hz, *J*_6,5_=9.0 Hz, *J*_7,5_=1.0, H_5_), 4.57 (1H, d, *J*=10.8 Hz, CH_2_Ph), 4.57 (1H, d, *J*_2,1_=3.6 Hz, H_1_), 4.67 (1H, d, *J*=12.1 Hz, CH_2_Ph), 4.70 (1H, ddd, *J*_8,8_=11.9 Hz, *J*_8,7_=5.4 Hz, *J*_8,6_=1.4 Hz, H_8_), 4.79 (1H, d, *J*=10.6 Hz, CH_2_Ph), 4.80 (1H, d, *J*=12.1 Hz, CH_2_Ph), 4.82 (1H, *J*=10.6 Hz, CH_2_Ph), 4.96 (1H, *J*=10.8 Hz, CH_2_Ph), 5.01 (1H, ddd, *J*_8,8_=11.9 Hz, *J*_8,7_=7.4 Hz, *J*_8,6_=1.4 Hz, H_8_), 5.61 (1H, dddd, *J*_7,6_=11.2 Hz, *J*_6,5_=9.0 Hz, *J*_8,6_=1.4 Hz, *J*_8,6_=1.4 Hz, H_6_) 5.81 (1H, dddd, *J*_7,6_=11.2 Hz, *J*_8,7_=7.4 Hz, *J*_8,7_=5.4 Hz, *J*_7,5_=1.0 Hz, H_7_), 7.22-7.37 (15H, m, Ph), 8.20 (1H, bs, NH); ^13^C-NMR: δ 55.5, 62.0, 66.8, 73.4, 75.3, 75.8, 79.8, 81.6, 81.9, 98.2, 127.6, 127.7, 127.7, 2x127.8, 127.9, 2x128.0, 2x128.1, 2x128.3, 2x128.4, 2x128.5, 131.2, 138.0, 138.1, 138.6, 157.3, 157.7; Anal. Calcd. for C_32_H_34_F_3_NO_6_ (585.63): C 65.63, H 5.85, N 2.39; found C 65.59, H 5.80, N 2.31.

*Methyl 2,3,4-tri-O-benzyl-6-[(trifluoroacetyl)amino]-7,8-dideoxy-d-glycero-α-d-galacto-oct-7-enpyranoside* **14a**, *Methyl 2,3,4-tri-O-benzyl-6-[(trifluoroacetyl)amino]-7,8-dideoxy-l-glycero-α-d-galacto-oct-7-enpyranoside* (**14b**): Following general procedure A, **13** (0.25 g, 0.43 mmol), K_2_CO_3_ (65.4 mg, 0.47 mmol) in *o*-xylene (2 mL) afforded after flash chromatography (hexane-ethyl acetate, 9:1) compounds **14a** and **14b** (0.08 g, 32%, see Table 4). Following general procedure B, **13** (0.10g, 0.171 mmol), K_2_CO_3_ (26 mg, 0.188 mmol) in *o*-xylene (2 mL) afforded after flash chromatography (hexane-ethyl acetate, 15:1) compounds **14a** and **14b** (0.07 g, 70%, see Table 2). 

**14a**: a colorless oil; [α]_D_^25^ = -19.6 **(***c* 0.23); ^1^H-NMR: δ 3.30 (1H, dd, *J*_5,4_=10.0 Hz, *J_4_*_,3_=9.2 Hz, H_5_), 3.33 (3H, s, OCH_3_), 3.49 (1H, dd, *J*_3,2_=9.3 Hz, *J*_2,1_=3.6 Hz, H_2_), 3.76 (1H, dd, *J*_5,4_=10.0 Hz, *J*_6,5_=1.4 Hz, H_5_), 4.01 (1H, dd, *J*_3,2_=9.3 Hz, *J*_4,3_=9.2 Hz, H_3_), 4.50 (1H, d, *J*=10.1 Hz, CH_2_Ph), 4.56 (1H, d, *J*_2,1_=3.6. Hz, H_1_), 4.66 (1H, d, *J*=12.1 Hz, CH_2_Ph), 4.82 (1H, d, *J*=12.1 Hz, CH_2_Ph), 4.84 (1H, d, *J*=10.7 Hz, CH_2_Ph), 4.90 (1H, d, *J*=10.1 Hz, CH_2_Ph), 4.97 (1H, ddd, *J*_6,NH_=9.4 Hz, *J*_7,6_=5.4 Hz, *J*_6,5_=1.4 Hz, H_6_), 5.01 (1H, d, *J*=10.7 Hz, CH_2_Ph), 5.23 (1H, dd, *J*_8*cis*,7_=10.3 Hz, *J_8cis,8trans_*=1.6 Hz, H_8*cis*_), 5.23 (1H, dd, *J*_8*trans*,7_=17.1 Hz, *J_8trans,8cis_*=1.6 Hz, H_8*trans*_), 5.81 (1H, ddd, *J*_8*trans*,7_=17.1 Hz, *J*_8*cis*,7_=10.3 Hz, *J*_7,6_=5.4 Hz, H_7_), 6.71 (1H, d, *J*_6,NH_=9.4 Hz, NH), 7.27-7.39 (15H, m, Ph); ^13^C-NMR: δ 50.8, 55.4, 71.0, 73.6, 75.6, 75.9, 78.0, 80.0, 81.8, 98.1, 117.2, 127.8, 2x128.0, 4x128.1, 2x128.4, 2x128.5, 4x128.6, 133.8, 137.4, 137.9, 138.2, 156.6, 157.0; Anal. Calcd. for C_32_H_34_F_3_NO_6_ (585.63): C 65.63, H 5.85, N 2.39; found C 65.56, H 5.79, N 2.32.

**14b**: a colorless oil; [α]_D_^25^ = +30.5 **(***c* 0.19); ^1^H-NMR: δ 3.35 (3H, s, OCH_3_), 3.41 (1H, dd, *J*_5,4_=10.0 Hz, *J*_4,3_=8.9 Hz, H_4_), 3.48 (1H, dd, *J*_3,2_=9.6 Hz, *J*_2,1_=3.5 Hz, H_2_), 3.81 (1H, dd, *J*_5,4_=10.0 Hz, *J*_6,5_=2.7 Hz, H_5_), 4.01 (1H, dd, *J*_3,2_=9.6 Hz, *J*_4,3_=8.9 Hz, H_3_), 4.57 (1H, *J*_2,1_=3.5 Hz, H_1_), 4.61 (1H, d, *J*=11.1 Hz, CH_2_Ph), 4.65 (1H, d, *J*=12.1 Hz, CH_2_Ph), 4.78 (1H, d, *J*=11.1 Hz, CH_2_Ph), 4.81 (1H, d, *J*=12.1 Hz, CH_2_Ph), 4.89 (1H, ddd, *J*_6,NH_=9.0 Hz, *J*_7,6_=8.2 Hz, *J*_6,5_=2.7 Hz, H_6_), 4.93 (1H, d, *J*=10.8 Hz, CH_2_Ph), 4.99 (1H, d, *J*=10.8 Hz, CH_2_Ph), 5.25 (1H, dd, *J*_8*trans*,7_=17.1 Hz, *J*_8*trans*,8*cis*_=1.0 Hz, H_8*trans*_), 5.29 (1H, dd, *J*_8*cis*,7_=10.3 Hz, *J*_8*trans*,8*cis*_=1.0 Hz, H_8*cis*_), 5.71 (1H, ddd, *J*_8*trans*,7_=17.1 Hz, *J*_8*cis*,7_=10.3 Hz, *J*_7,6_=8.2 Hz, H_7_), 6.70 (1H, d, *J*_6,NH_=9.0 Hz, NH), 7.27-7.39 (15H, m, Ph); ^13^C-NMR: δ 52.4, 55.5, 71.6, 73.8, 74.7, 76.0, 77.8, 80.1, 82.1, 98.5, 121.4, 2x127.8, 128.0, 128.1, 2x128.2, 3x128.3, 2x128.7, 4x128.8, 131.1, 2x138.1, 138.6, 156.3, 156.7; Anal. Calcd. for C_32_H_34_F_3_NO_6_ (585.63): C 65.63, H 5.85, N 2.39; found C 65.53, H 5.76, N 2.45
